# Bacterial contamination of irrigation fluid and suture material during ACL reconstruction and meniscus surgery: Low infection rate despite increasing contamination over surgery time

**DOI:** 10.1007/s00167-021-06481-3

**Published:** 2021-02-10

**Authors:** Benjamin Bartek, Tobias Winkler, Anja Garbe, Tarek Schelberger, Carsten Perka, Tobias Jung

**Affiliations:** 1 Center for Musculoskeletal Surgery, Charitéplatz 1, 10117 Berlin, Germany; 2 Julius Wolff Institute, Berlin, Germany; 3 Berlin-Institute of Health Center for Regenerative Therapies, Charité, Universitätsmedizin Berlin, Corporate Member of Freie Universität Berlin, Humboldt-Universität Zu Berlin, and Berlin Institute of Health, Berlin, Germany

**Keywords:** ACL reconstruction, Meniscus surgery, Infection, Contamination, Prophylaxis

## Abstract

**Purpose:**

During knee arthroscopy, irrigation fluid from the surgical site accumulates in the sterile reservoir. Whether these fluid collections and also suture material used during the arthroscopic surgical processes show bacterial contamination over time during surgery remains unclear. The purpose of this study was to determine this contamination rate and to analyze its possible influence on postoperative infection.

**Materials and methods:**

In this study, 155 patients were included. Fifty-eight underwent reconstruction of the anterior cruciate ligament (ACL), 63 meniscal surgery and 34 patients combined ACL reconstruction and meniscus repair. We collected pooled samples of irrigation fluid from the reservoir on the sterile drape every 15 min during the surgery. In addition, we evaluated suture material of ACL graft and meniscus repair for bacterial contamination. Samples were sent for microbiological analysis, incubation time was 14 days.

All patients were seen in the outpatient department 6, 12 weeks and 12 months postoperatively and examined for clinical signs of infection.

**Results:**

A strong statistical correlation (*R*^2^ = 0.81, *p* = 0.015) was found between an advanced duration of surgery and the number of positive microbiological findings in the accumulated fluid. Suture and fixation material showed a contamination rate of 28.4% (29 cases). Despite the high contamination rate, only one infection was found in the follow-up examinations, caused by *Staphylococcus lugdunensis*.

**Conclusion:**

Since bacterial contamination of accumulated fluid increases over time the contact with the fluid reservoirs should be avoided.

**Level of evidence:**

IV.

## Introduction

Although knee arthroscopy is generally considered an effective and safe procedure, it is not free from complications [14]. Among these, postoperative joint infections are rare but severe adverse events with an overall incidence of less than 1%. Comparing different arthroscopic procedures, with an incidence ranging from 0.3 to 1.7%, ACL reconstruction is responsible for postoperative infection more often than, e.g. meniscus surgery (incidence 0.15–0.84%) [1, 7, 11]. This study demonstrates that time-dependent contamination of irrigation fluid and bacterium stained suture material are key determinants in post-arthroscopic site infection after ACL reconstruction and meniscus repair. In contrast to other potentially contagious factors, as listed in the national nosocomial infections surveillance score (NNIS), fluid and suture contamination is an avoidable risk and knowledge about their impact, therefore, a key factor in the prevention of postoperative adverse events.

Risk factors for joint infection after surgery can be divided into surgery related and patient related. Smoking and obesity, for example, are known to be major positive predictors concerning deep and superficial infection. On the procedural side, e.g. complexity and duration of the surgical procedure are associated with a higher risk of postoperative infection [11].

To prevent joint infection, orthopedic surgery requires a highly sterile environment since the joint is a secluded compartment with a scarcity of immune cells [20]. Despite additive preventive measures such as single-shot antibiotic prophylaxis or laminar airflow, the risk for septic arthritis after arthroscopic knee surgery remains up to 1.1% [6, 19].

The continuous flush with a sterile solution, which is inherent to arthroscopic surgery, is one basic measure for the intraoperative prophylaxis against infection. In animal models, intensified lavage of contaminated wounds achieved a clear reduction in microbial load [21]. However, fluid residues accumulating from the remains of irrigation fluid, blood and debris on the surgical drape also provide a potential reservoir for pathogenic bacteria [2]. Suction devices, commonly used to remove accumulating irrigation fluid, also showed substantial bacterial contamination during total knee arthroplasty [15].

To what extent the even bigger fluid reservoirs in arthroscopic procedures remain sterile over the course of surgery or whether there is a time-dependent increase in their contamination has not been investigated so far. Therefore, the objective of the current study was to investigate whether pathogenic microbes can be identified in the irrigation fluid of arthroscopic ACL and meniscus surgery and to determine potential operation time thresholds for increased fluid contamination.

Our hypothesis was that despite the continuous flush in arthroscopic surgery, an increasing rate of microbiological contamination of the irrigation fluid and suture material over time would be observed. Respective to fluid, this has already been shown for joint replacement surgery, but has not yet been examined for knee arthroscopies. Suture material has not been evaluated in this context yet, despite its critical role in arthroscopic procedures.

## Materials and methods

The study was approved by the institutional review board of the Charité, Universitätsmedizin Berlin (EA4/069/17). All patients gave written informed consent prior to study inclusion.

### Patients and microbiological analysis

In this prospective, single-center study, 155 patients of which 58 underwent reconstruction of the ACL and 63 meniscal surgery were included. Thirty-four patients underwent combined ACL reconstruction and meniscus repair. The inclusion criteria were a minimum age of 18 years and anterior cruciate ligament rupture or meniscal injury proven by MRI and clinical examination. A written informed consent was obtained from patients to participate in the study. The exclusion criteria were any previous knee surgery or joint infection, skin abrasions, inflammatory skin diseases such as psoriasis and neurodermatitis and body-mass-index above 30 as well as nicotine abuse. Two experienced, high volume surgeons performed the procedures between March and June 2018. The operation room was equipped with a laminar airflow system and outwardly directed excess pressure. The surgeons wore standard sterile disposable operation room clothing and two pairs of surgical gloves. Patients received single-shot perioperative antibiotic prophylaxis (Cefazolin 2 g intravenously 30 min before skin incision). In the event of cephalosporin intolerance, 1 g of vancomycin was administered intravenously 2 h before skin incision. All patients underwent shaving of the surgical site with a disposable razor immediately before surgery.

Standard pre-cleaning of the patient’s lower extremity was performed using an alcoholic solution (Softasept N, B. Braun) before final disinfection. Following this, the skin was disinfected four times with a povidone–iodine solution (Braunol, B. Braun) for at least 10 min in total. Sterile draping was performed following a routine protocol, with a two-layer sterile covering drape including one reservoir.

Purisole^®^ solution (5000 ml, Fresenius Kabi) was used as the irrigation solution. Before each surgery, one fluid sample was taken under sterile conditions using a syringe with cannula directly from the irrigation bag to exclude preexisting fluid contamination. After defined time intervals of 15 min each, 5 ml of irrigation fluid was collected from the reservoir of the sterile surgical drape using new syringes at all time points and injected into sterile tubes. In addition, during surgery, all suture and fixation material previously used for meniscus repair [2–0, non-absorbable, UHMW polyethylene ULTRABRAID™ Suture (Fast-Fix 360, Smith and Nephew); 2–0 and 0, absorbable, polydioxanon suture (Ethicon)] or tendon preparation [non-absorbable polyester fibers (PremiCron^®^, B. Braun)] was collected in sterile tubes immediately after use for subsequent microbiological testing. In case of partial meniscectomy, only fluid samples were investigated.

All samples were sent to the microbiology institute, where they were incubated for 14 days. In case of positive microbiological results, bacterial species were identified and resistograms established. This experimental design was more susceptible to false-negative than to false-positive results as all fluid samples were taken from a relative big reservoir with high chances of missing the overall small bacterial load and its uneven distribution. Therefore, to prevent the underestimation of contamination, cases, in which specimen from one and the same patient showed negative microbiological results after a previous culture-positive microbiological finding, were rated as false negative.

A soft wound drain (BLAKE Silicone Drain^®^, Ethicon) was used only in patients who underwent ACL reconstruction. No postoperative antibiotics were given in any of the evaluated cases.

All patients were seen in the outpatient department 6 and 12 weeks postoperatively and examined for clinical signs of infection. 12 months after surgery, a telephone consultation was performed inviting each patient to join final follow-up evaluation at our outpatient clinic. If the patient failed to appear personally, a questionnaire-based telephone interview was conducted to inquire patient satisfaction with surgery and to evaluate knee function and condition, also evaluating the possibility of a possible infection.

### Statistical analysis

Statistical analysis was performed to evaluate the correlation between the duration of the surgery and the rate of positive microbiological findings. As the respective data were not normally distributed, Spearman's rank correlation coefficient for non-parametric variables was used. The two-tailed significance level was set to *p* = 0.05. As no comparable study had been published before, no data were available for an a priori power analysis. However, a post hoc power analysis based on the parameters of this study (*R*^2^ = 0.81, *p* = 0.015 and *n* = 155) was conducted and confirmed a very good power of > 0.99 for the calculation of a two-tailed bivariate correlation.

## Results

The patient population comprised 67 women and 88 men with an average age of 33.7 years (SD, 12.6 years). The average duration for ACL reconstruction was 65 min, for meniscus surgery 36 min and for ACL reconstruction and meniscus repair 75 min.

### Irrigation fluid

**Fig. 1:**
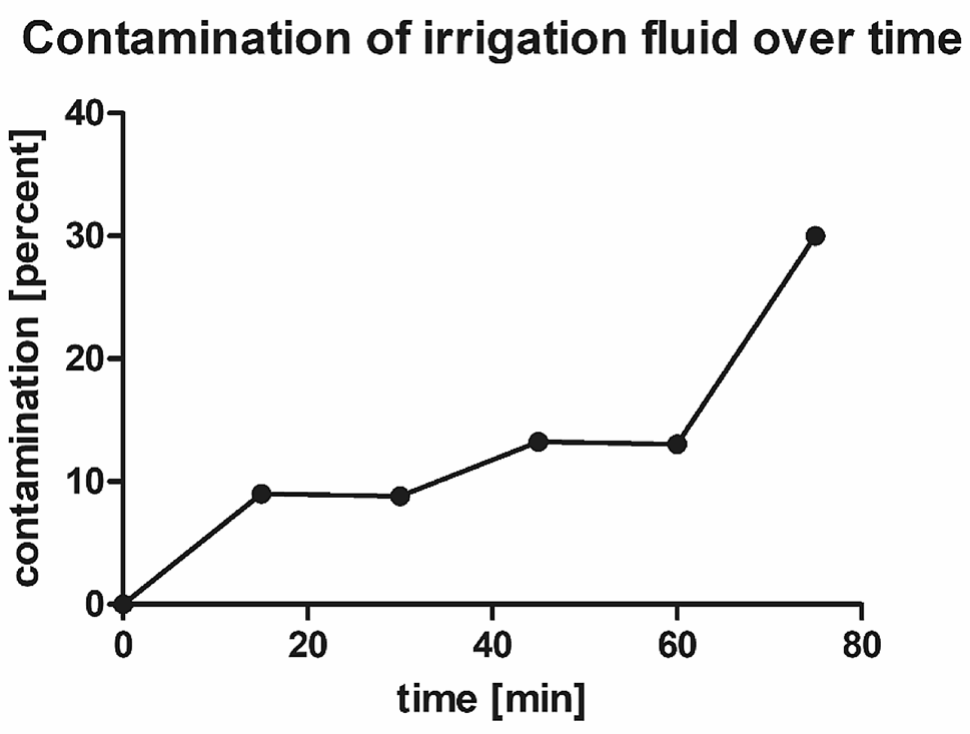
Graph showing positive bacterial cultures of the surgical cover; percentage of microbiologically positively tested samples from irrigation fluid depending on time

No samples of the fluid bags were contaminated prior to surgery. The contamination rate of collected irrigation fluid increased over time and peaked to 30.0% after 75 min (Fig. 1).

Accordingly, a very strong statistical correlation (*R*^2^ = 0.81, *p* = 0.015) was found between an advanced duration of surgery and the number of positive microbiological findings. Out of the total cohort of 155 patients, 29 patients (18.7%) showed at least one culture-positive finding in their irrigation fluid samples.

Of the total number (*n* = 536) of samples studied, 40 samples were positive (7.5%) (Table 1).

**Table 1:** Microbial identification in irrigation fluid

	Total irrigation fluid contamination [*n* (%)]
*Staphylococcus epidermidis*	12 (36.4)
*Cutibacterium acnes*	9 (27.3)
*Staphylococcus capitis*	6 (18.2)
*Staphylococcus lugdunensis*	2 (7.1)
*Micrococcus luteus*	1 (3)
*Paenibacillus peoriae*	1 (3)
*Staphylococcus hominis*	1 (3)
*Staphylococcus aureus*	1 (3)

Numbers and percentage of specific bacteria in all positive specimens

Regarding the type of surgery, positive microbiological findings of irrigation fluid during isolated ACL reconstruction occurred in 19.0% (*n* = 11/58). The contamination rate of irrigation fluid was 12.7% (*n* = 8/63) during isolated meniscus surgery and 29.4% (*n* = 10/34) during ACL reconstruction combined with meniscal repair.

### Suture and fixation material

**Fig. 2:**
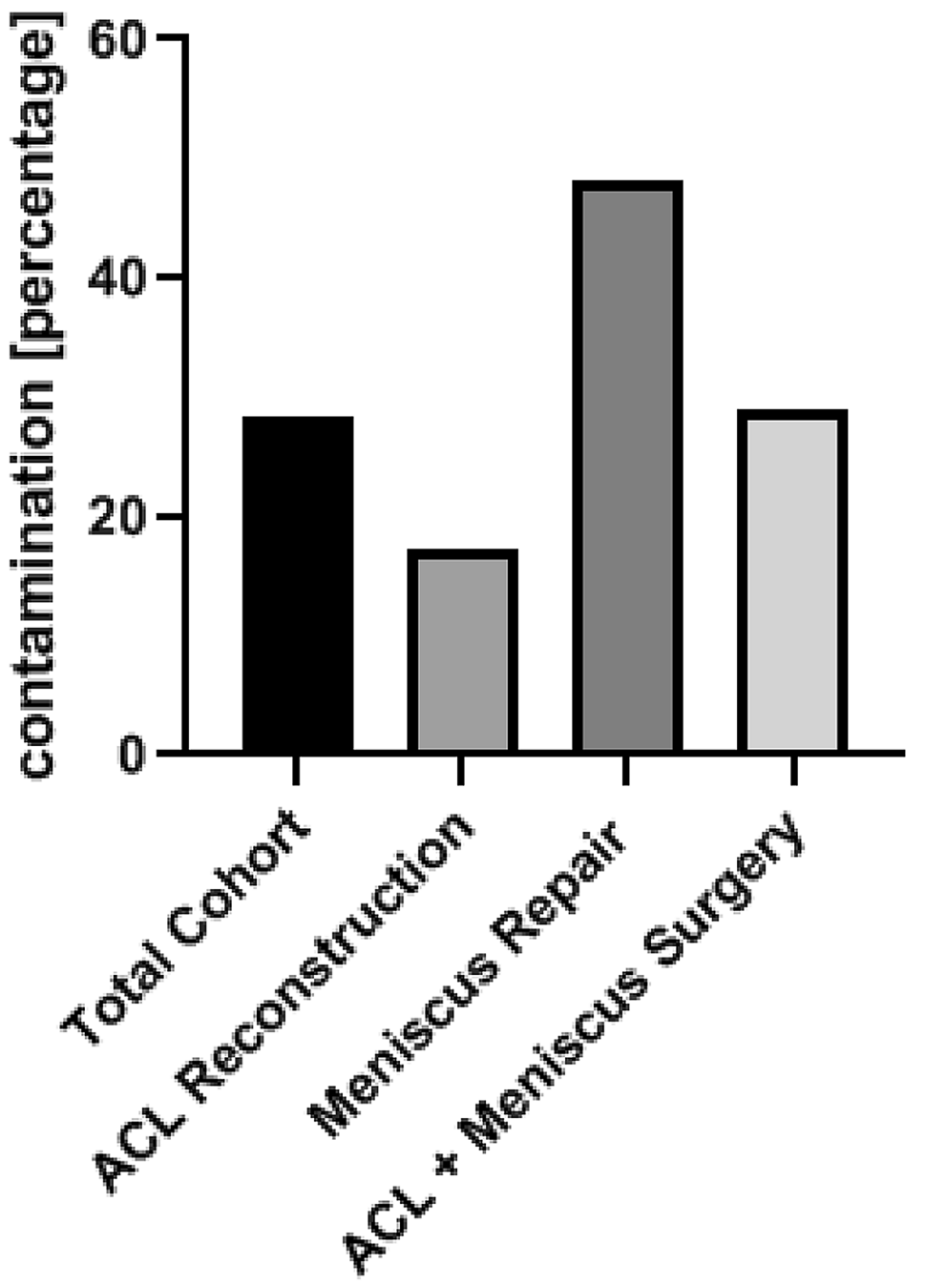
Graph showing positive bacterial cultures. For isolated ACL reconstruction, positive microbiological cultures occurred in 17.4% (*n* = 8/46 cases) of suture and fixation material. Patients undergoing meniscus repair showed the contamination rates of 48.0% (*n* = 12/25 cases) of suture and fixation material. For ACL reconstruction combined with meniscal repair, the contamination rate was 29.0% (*n* = 9/31 cases) of suture and fixation material

Suture and fixation material of 102 patients (58 male, 44 female) was examined, which showed a contamination rate of 28.4% (29 cases) (Fig. 2). The detected bacteria on suture and fixation material vary in the different types of surgery (Table 2).

**Table 2:** Microbial identification on suture material for each group

	ACL reconstruction [*n* (%)]	Meniscus repair [*n* (%)]	ACL + meniscus surgery [*n* (%)]
*Staphylococcus epidermidis*	2 (22.2)	4 (30.8)	4 (36.4)
*Cutibacterium acnes*	5 (55.6)	4 (30.8)	
*Staphylococcus capitis*		1 (7.7)	
*Staphylococcus lugdunensis*	1 (11.1)		1 (9.1)
*Micrococcus luteus*			2 (18.2)
*Bacillus licheniformis*	1 (11.1)		
*Staphylococcus hominis*		1 (7.7)	2 (18.2)
*Staphylococcus cohnii*			1 (9.1)
*Staphylococcus saccharolyticus*		1 (7.7)	
*Staphylococcus haemolyticus*		1 (7.7)	
*Staphylococcus parasanguinis*		1 (7.7)	
*Staphylococcus lentus*			1 (9.1)

Within the 29 cases with culture-positive findings of the total cohort (*n* = 155), bacterial contamination was found in both, irrigation fluid and suture material, in 8 cases (27.6%).

**Fig. 3:**
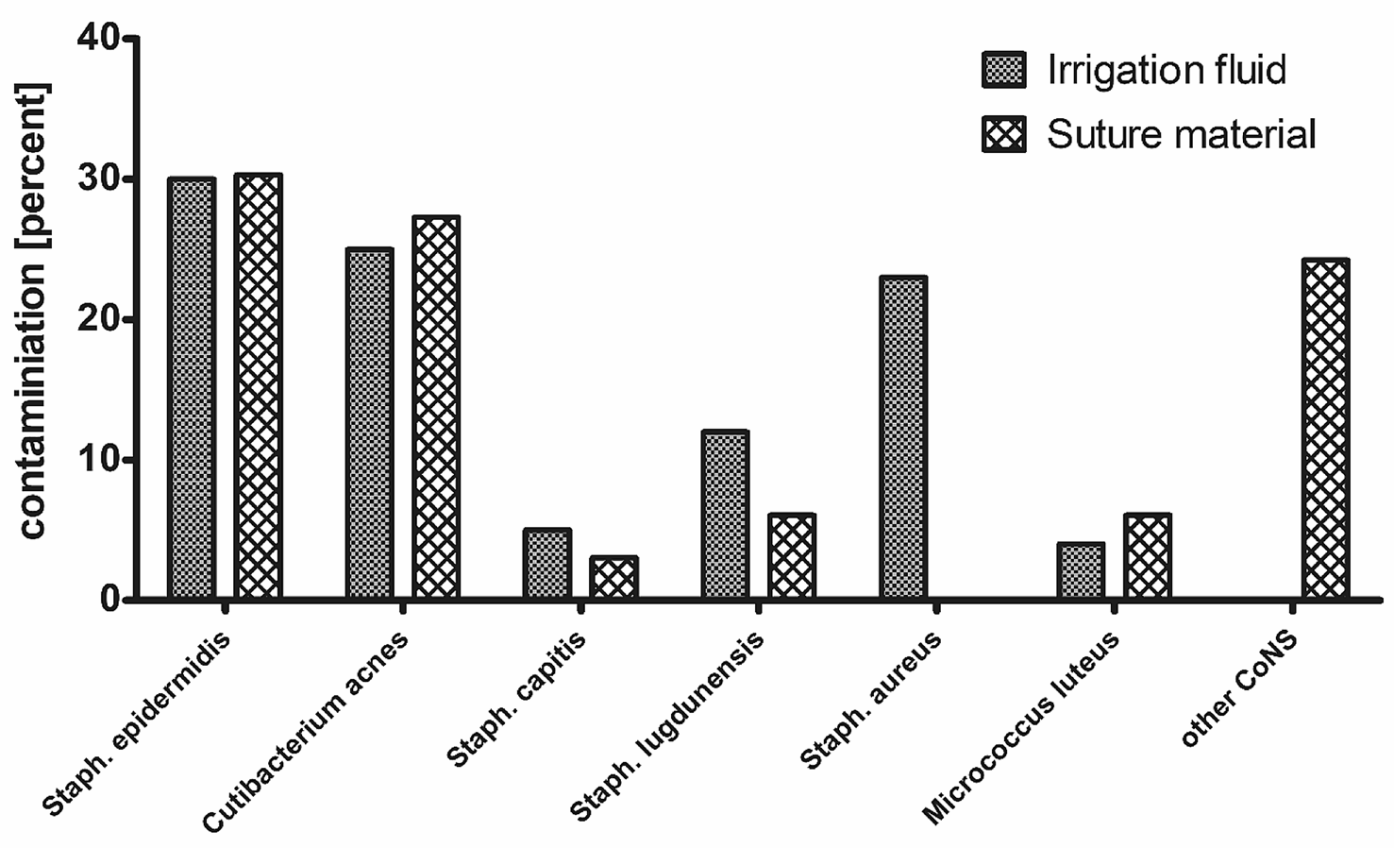
Graph showing microbial identification; percentage of microbial differentiation of all positive final microbiological findings. *CoNS* coagulase-negative staphylococci

Overall, the most frequent pathogen was *Staphylococcus epidermidis* and the second most frequent was *Cutibacterium acnes* (Fig. 3).

Over the follow-up period of 12 months, intra-articular hematoma developed in two patients, which did not require surgical intervention within the follow-up period. Only one clinically and laboratory proven postoperative joint infection was detected in the total patient cohort. This patient received an isolated ACL reconstruction and presented in our emergency department with pain, joint redness and fever 6 days after surgery. In the revision surgery (arthroscopic synovectomy, irrigation and debridement), *Staphylococcus lugdunensis* was identified, which was also isolated previously from both irrigation fluid and suture samples of the initial ACL reconstruction surgery.

## Discussion

The most important finding of this study was a surgical time-dependent contamination of irrigation fluid and suture material in ACL reconstruction and meniscus repair. We are not aware of any comparative studies on this subject with respect to knee arthroscopy.

Our data show for the first time that in arthroscopic knee surgery irrigation fluid reservoirs on surgical covers are prone to increasing bacterial contamination over time. Furthermore, as postoperative knee infection is attributed to a multitude of different microorganisms, which are predominantly part of the physiological skin flora [12, 22], bacterial contamination of irrigation fluid and suture and fixation material first investigated in this study may not necessarily imply, but could be the potential source of surgical site infection.

To the best of our knowledge, this study is the first to show the progression of contamination over time at short intervals and to compare the pathogen colonization of suture material with the colonization of the irrigation fluid. Gowd et al. showed operative time to be an independent risk factor for complications including surgical site infection in knee arthroscopy without further investigating its underlying mechanisms [17]. Fuchs et al. demonstrated a contamination rate of 22% for fluid residues in knee arthroplasty after 60 min of surgery [15]. Pauzenberg et al. showed increased skin contamination at the end of shoulder arthroscopy despite perioperative preventive measures. At the end of shoulder arthroscopy, 64.6% of patients showed at least one culture positive for *C. acnes* [22].

Only few studies have yet dealt with the contamination of suture material in arthroscopic surgery [23, 26]. Roach et al. showed a relevant microbial colonization of subscapularis tagging sutures in shoulder surgery and again a significant association of procedure length and positive microbacterial finding (*p* = 0.03) [23]. There is no comparable study for the knee joint in the literature.

As there was only one postoperative joint infection in this series, no final assumption can be made whether positive suture or irrigation fluid culture is a more reliable factor for predicting the risk of surgical site infection. The reported infection was a *Staphylococcus lugdunensis* infection after positive fluid and suture specimens for this bacterium. This example indicates that in case of bacteria with high virulence such as *Staphylococcus aureus* or intermediate virulence such as *S. lugdunensis*, the contamination of the material and fluid can be relevant. However, the majority of contaminations did not lead to a postoperative infection. This might be due to the character of the arthroscopic surgery with continuous flushing of the operation site and the standard-of-care single-shot antibiotic, which might be sufficient in most cases to fight the evolvement of an infection.

Interestingly enough, only one out of four patients with same positive suture material and irrigation fluid results (only 0.6% of all patients and 2% of all culture-positive patients examined) developed a postoperative knee infection during the follow-up period. All other culture-positive patients remained free of any sign of infection in our 12-month follow-up.

The pathogen causing the only postoperative joint infection in this study, *Staphylococcus lugdunensis*, is involved in a wide range of severe infections. Frank et al. were the first to show *S. lugdunensis* to be a virulent CoNS that behaves similarly to *Staphylococcus aureus* in causing deep abscesses, skin and soft tissue infections, central nervous system infections and blood stream infections including sepsis, toxic shock syndrome and endocarditis complicated by embolic events [5, 18]. With its specific adhesions molecules, cytotoxins and biofilm synthesis system, *S. lugdunensis*, although a CoNS, shows a high pathogenicity comparable to *S. aureus* and is frequently verified in post-arthroscopic joint infections [3]. Therefore, single positive microbiological samples with *S. lugdunensis* should be treated as significant rather than as contamination [4, 13].

A discrepancy between high bacterial contamination rates and a low rate of postoperative infection was noticed. First, it is possible that most potential infections in those patients were successfully prevented by perioperative antibiotic prophylaxis [10, 24]. Second, the continuous flush of the knee joint during arthroscopic surgery leads to pathogen elimination in the case of a (low) bacterial contamination load. Third, there may be some yet unknown patient-related endogenous immunologic capacities, which enable patients to contain a certain number of pathogens inducing postoperative infections. Additional studies are surely necessary to clarify these questions.

Graft preparation with vancomycin is a well-known and investigated procedure to decrease infection after ACL reconstruction [10]. This proven concept might also be applied for all used suture and fixation material to reduce bacterial contamination and need to be evaluated in further studies.

Despite the efforts for a rigorous methodology, there are limitations to this study. Only one sample was examined at the respective times and thus contamination with a large dilution may not have been detected. Furthermore, all patients in this study received a single-shot perioperative cefazolin prophylaxis (or vancomycin in the event of cephalosporin intolerance) so that all samples were taken after administration of antibiotics. Although known from arthroplasty and periprosthetic joint infection studies that a single-shot antibiotic does not compromise the result of intra-articular biopsy [8, 9, 16, 25], this could have led to a certain selection phenomenon. Nevertheless, antibiotic prophylaxis is a common measure and is used as standard of care in everyday clinical practice. In addition, the relatively short follow-up period of 12 months may have not allowed to detect all, particularly low-grade infections. Therefore, the true rate of joint infection could be higher than the one we observed. To the best of our knowledge, the present study is the first one examining the correlation between the duration of the surgery and contamination rates of the pooled irrigation fluid. Thus, no data for a reliable a priori power analysis were available and we, therefore, have conducted the study in an explorative manner.

The data showed only one case of a postoperative infection accompanied by suture and irrigation fluid contamination. Nevertheless, the time-dependent increase of suture and fluid contamination should contribute to a more distinct awareness in the handling of these potentially contaminated materials during surgery, e.g. contact of surgical instruments with the fluid reservoir should be avoided as should be emptying it using the suction device.

## Conclusion

Any direct contact with the collected irrigation fluid in the reservoir and any contact of suture material with skin or fluid reservoirs should be avoided.
